# Safety and Immunogenicity of Early Bacillus Calmette-Guérin Vaccination in Infants Who Are Preterm and/or Have Low Birth Weights: A Systematic Review and Meta-analysis

**DOI:** 10.1001/jamapediatrics.2018.4038

**Published:** 2018-11-26

**Authors:** Shiraz Badurdeen, Andrew Marshall, Hazel Daish, Mark Hatherill, James A. Berkley

**Affiliations:** 1 Department of Paediatrics, University of Oxford, United Kingdom; 2 Newborn Research Centre, The Royal Women's Hospital, Parkville, Victoria, Australia; 3 Children’s Hospital, John Radcliffe Hospital, Headington, Oxford, United Kingdom; 4 Children’s Services, Oxford University Hospitals National Health Services Foundation Trust, Headington, Oxford, United Kingdom; 5 Department of Paediatrics, Chelsea and Westminster Hospital National Health Services Trust, Chelsea, London, United Kingdom; 6 South African Tuberculosis Vaccine Initiative, Institute of Infectious Disease and Molecular Medicine and Division of Immunology, Department of Pathology, University of Cape Town, Cape Town, South Africa; 7 KEMRI/Wellcome Trust Research Programme, Kilifi, Kenya; 8 The Childhood Acute Illness & Nutrition Network, Nairobi, Kenya; 9 Centre for Tropical Medicine & Global Health, University of Oxford, Oxford, United Kingdom

## Abstract

**Importance:**

Bacillus Calmette-Guérin (BCG) vaccination is commonly delayed in infants who are preterm and have low birth weights (LBW) despite the association of early vaccination with better vaccination coverage and potentially nonspecific benefits for survival.

**Objective:**

To determine the safety, immunogenicity, and protective efficacy against tuberculosis (TB) of BCG vaccination given at or before 7 days after birth vs vaccination more than 7 days after birth among infants who are preterm and/or had LBW.

**Data Sources:**

Searches of Medline, Embase, and Global Health databases were conducted from inception until August 8, 2017.

**Study Selection:**

Clinical trials, cohort studies, and case-control studies that included infants who were preterm and/or had LBW and reported safety, mortality, immunogenicity, proxies of vaccine take, and/or efficacy against TB.

**Data Extraction and Synthesis:**

Two authors independently extracted data and assessed the quality of the studies. Data extracted included demographics, covariates, sources of bias, and effect estimates. Meta-analysis was performed using a random-effects model.

**Main Outcomes and Measures:**

Safety, mortality, immunogenicity, or other proxies of vaccine take, such as tuberculin skin test (TST) conversion and efficacy against tuberculosis.

**Results:**

Forty studies were included in a qualitative synthesis; infants who were preterm (born at 26-37 weeks’ gestational age) and/or had LBW (0.69-2.5 kg at birth) were included. The BCG vaccine was administered at or before 7 days to 10 568 clinically stable infants who were preterm and/or had LBW; vaccination was administered to 4310 infants at varying times between 8 days and 12 months after birth. Twenty-one studies reporting safety found no cases of BCG-associated death or systemic disease in 8243 infants. Four studies reported no increase in all-cause mortality for infants who had LBW and who received early BCG vaccination compared with infants who had LBW with later vaccination or BCG-vaccinated infants of normal birth weight. Four studies reported lymphadenitis incidence; combined, these reported 0% to 2.9% incidence of vaccination within 7 days and 0% to 4.2% of vaccination after 7 days. Meta-analysis of 7 studies revealed no differences between early and delayed BCG vaccination for scar formation (n = 515; relative risk [RR], 1.01 [95% CI, 0.95-1.07]) or TST conversion (n = 397; RR, 0.97 [95% CI, 0.84-1.13]). Published data were insufficient to assess immunogenicity or protective efficacy against TB disease.

**Conclusions and Relevance:**

Early BCG vaccination in healthy infants who are preterm and/or had LBW has a similar safety profile, reactogenicity, and TST conversion rate as delayed vaccination. Based on current evidence, early BCG vaccination in stable infants who are preterm and/or have LBW to optimize uptake is warranted.

## Key Points

**Question:**

Is administration of bacillus Calmette-Guérin (BCG) vaccination within 7 days of birth safe, immunogenic, and efficacious in infants who are preterm and/or have low birth weight, compared with BCG vaccination at later points or in infants who are normal birth weight?

**Findings:**

In this systematic review and meta-analysis, we found no increase in adverse reactions or infant mortality after BCG vaccination within 7 days of birth compared with vaccination delayed after 7 days in clinically stable infants who were preterm and/or had low birth weight. Meta-analysis revealed no differences for scar formation or tuberculin skin test conversion.

**Meaning:**

Currently, evidence from clinically stable infants who were born after more than 30 weeks’ gestational age and/or weighing more than 1.5 kg seems to support BCG vaccination within 7 days of birth.

## Teaser

This systematic review and meta-analysis examines the safety and efficacy of bacillus Calmette-Guérin vaccination at birth or later points in infants who are preterm and/or had low birth weights compared with ones who are full term and/or had normal birth weights.

## Introduction

Bacillus Calmette-Guérin (BCG) is the most widely administered vaccine worldwide.^1^ It remains the only approved vaccine for the prevention of tuberculosis (TB), which globally affects more than 10 million individuals annually.^2^ The BCG vaccine is usually given soon after birth to infants who were full term and reduces incidence of TB disease and TB-associated mortality in childhood.^3,4^ However, among the 15 million infants born preterm and 20 million born with low birth weight (LBW) each year worldwide, BCG is commonly delayed because of uncertainty about safety and immunogenicity.^5,6^ International and national guidelines are inconsistent on the timing of BCG vaccination in infants who are preterm and/or LBW, ranging between early vaccination and deferring BCG until discharge from hospital or at a follow-up appointment. The most recent World Health Organization (WHO) position paper highlights the lack of systematic evidence on safety and efficacy, particularly for infants with LBW.^7,8,9,10,11^

Delayed administration of BCG has been reported to be associated with reduced vaccination coverage and equitability.^12,13,14,15^ Two large, recent cohort studies from Ghana and Kenya showed that LBW was an independent risk factor for BCG vaccination delay, with 25% to 60% nonvaccination in the first 4 weeks of life and ongoing slow uptake thereafter.^12,13^ The observation that BCG vaccination is more delayed among home-born infants who have LBW suggests that parental concern about the infant’s fragility may be a contributory factor.^12,16^ The current global vaccination action plan emphasizes increasing coverage and equitability by targeting groups underserved by current strategies.^17^ Administering BCG vaccine shortly after birth, when the family is engaged with health care professionals, is logistically straightforward; however the safety, immunogenicity, and protective efficacy of BCG vaccine against TB is unclear in this population.

Disseminated BCG disease is a potentially fatal complication among individuals who are immunocompromised, and the WHO advises against BCG vaccination of known HIV-infected infants.^18,19^ Suppurative BCG lymphadenitis is more common in neonates than older children, and risk is limited by dose reduction.^19^ The concept that infants who are preterm and/or have LBW are immunologically immature prompts uncertainty about the risks of both morbidity and protective immunogenicity among these infants if they are vaccinated at birth.^20,21,22,23^ It has been suggested that delaying BCG vaccination in infants who are full term may result in an enhanced memory CD4 T-cell response; some studies on this topic have been inconsistent.^24,25,26^

There is also increasing interest in potential heterologous effects of early BCG vaccination, reported to be associated with reduced all-cause mortality that cannot be explained by protection against TB alone.^27^ The WHO has appealed for further evidence before modifying the Expanded Programme of Immunization recommendations.^11^ We aimed to systematically review the published literature to address the following clinical question: in infants who are preterm and/or have LBW, is early BCG vaccination safe, immunogenic, and efficacious compared with delayed BCG vaccination?

## Methods

### Search Strategy and Selection Criteria

This meta-analysis is presented according to Preferred Reporting Items for Systematic Reviews and Meta-analyses (PRISMA) guidelines.^28^ Medline, Embase, and Global Health databases were searched using the Ovid interface without language or date restrictions. Search terms included multiple variants of terms *BCG*, *prematurity*, *low-birth weight*, and *small for gestational age*, additional to Medical Subject (MeSH) Headings (eMethods in the Supplement). Randomized clinical trials (RCTs) of BCG vaccination in newborns (without terms limiting the included population by gestational age or birth weight) were also searched. Snowball searching was performed by screening the references of retrieved studies. Experts in the field were contacted. The search was last updated on August 8, 2017.

Predefined inclusion criteria were randomized clinical trials, cohort studies, or case-control studies that (1) included BCG vaccination of neonates who were preterm (those born at <37 weeks’ gestational age), LBW (born weighing <2.5 kg), or both, and (2) reported safety, mortality, immunogenicity, measures of vaccine take (such as tuberculin skin test [TST] conversion), or efficacy against TB disease. Studies were included if they compared BCG vaccination in infants who were preterm and/or LBW and less than or equal to 7 days old vs those more than 7 days old, infants who were preterm vs full term, or infants with LBW vs those with normal birth weights (NBW). Including the second and third analyses allowed comparison with infants who were full term and/or had NBWs as a reference population for safety, immunogenicity, and efficacy.

We examined safety data on local and regional reactogenicity (eg, erythema, induration, ulceration, scarring, abscess formation, cutaneous lesions, and lymphadenitis); systemic adverse reactions (eg, osteomyelitis and disseminated BCG disease); mortality; measures of immunogenicity, including T-cell responses to purified protein derivative (PPD), BCG, or specific *Mycobacterium tuberculosis* antigens or antigen pools; reported vaccine take assessments including TST conversion; and protective efficacy against TB disease.

### Screening and Data Extraction

We excluded studies of infants who were full term and/or NBW only, studies without specific outcome data on infants who were preterm or LBW, studies with duplicate data, and studies reporting only responses to heterologous non-Mycobacterial antigens. After removal of ineligible studies and duplicates, 2 authors (S.B. and A.M.) independently screened all titles and abstracts. If either reviewer thought an abstract may meet inclusion criteria, the full text of the article was reviewed. Discrepancies were resolved by the senior author (J.A.B.). Data were extracted into tables that included study location; design; methods of recruitment; sample size; population characteristics; age at vaccination; BCG strain; methods used for measuring outcomes; and results on safety, immunogenicity, TST conversion, and protective efficacy against TB. Where indicated, study authors were contacted to request additional data or clarification as needed.

### Risk of Bias Assessment

Two independent reviewers (S.B. and H.D.) assessed the included studies using tools from the Cochrane collaboration.^29,30^ For domains with insufficient information for adequate assessment risk of bias (such as undescribed randomization procedures), and where this domain was likely to affect the outcome of interest, a judgement of high risk was made. For studies presenting safety alone, methodological quality assessment was restricted to this outcome.

### Statistical Analysis

For studies comparing infants who were preterm and/or had LBW and were vaccinated at birth with those who experienced vaccination at later points, we performed meta-analyses using random-effects models. We used the Mantel-Haenszel method for dichotomous data and presented the results as relative risks (RR) with 95% CIs. Statistical heterogeneity was assessed using the *I^2^* test for each outcome. Sensitivity analyses were planned and performed for assessing the effects of heterogeneous studies and studies with high risk of bias by sequential exclusion. Meta-analysis was performed using Review Manager (RevMan) version 5.3 (Cochrane Collaboration).

## Results

**Figure 1.**
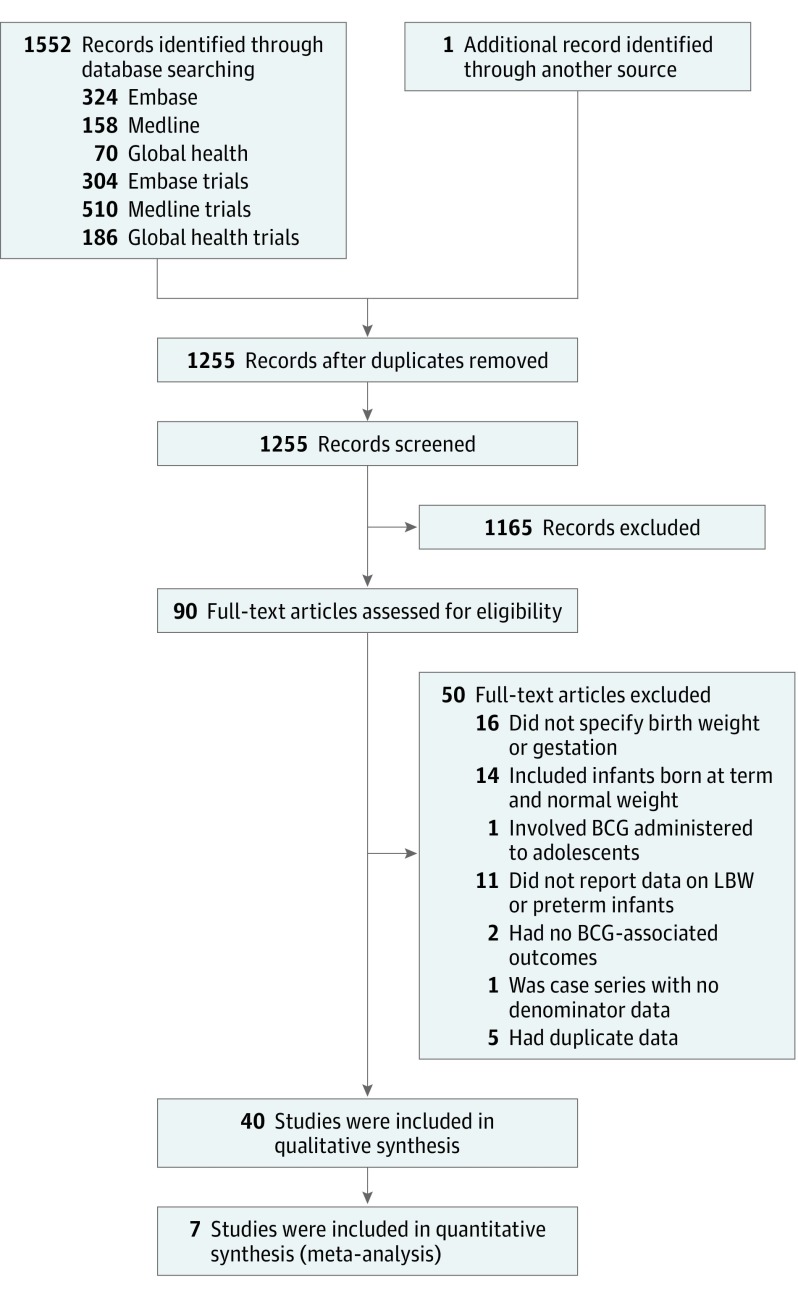
Flow Diagram of Study Selection BCG indicates bacillus Calmette-Guérin vaccine; LBW, low birth weight.

The search returned 1552 articles. After removal of duplicate data and screening, 40 articles were identified (Figure 1).^14,20,21,22,23,31,32,33,34,35,36,37,38,39,40,41,42,43,44,45,46,47,48,49,50,51,52,53,54,55,56,57,58,59,60,61,62,63,64,65^ Most studies were prospective cohorts or randomized clinical trials, conducted predominantly between 1990 and 2017 in Africa (k = 14 [where *k* indicates the number of studies]), Asia (k = 10), Europe (k = 8), South America (k = 5) and the Middle East (k = 3). One study included 2019 infants who were preterm and 2419 newborns who had LBW who had received early BCG vaccination but did not report safety outcomes independently from infants who were full term and/or had NBWs. In this case, individual patient data were obtained for post hoc analysis.^48^

Overall, BCG vaccination was administered to 10 568 clinically stable neonates who were preterm and/or had LBW and aged 7 days or younger. Delayed vaccination was administered to 4310 infants at 8 days to 12 months after birth. The most commonly reported vaccination points were when the infant was near full term gestational age, had reached a weight of more than 2 kg or 2.5 kg, or was discharged from the hospital. Almost all infants were vaccinated by 6 months after birth. Gestational ages for infants who were preterm ranged from 26 to 37 weeks, and birth weights for infants identified as having LBW ranged from 0.69 kg to 2.5 kg.

### Risk of Bias Assessment

Most studies were of moderate or high risk of bias. The most common causes of potential bias were lack of assessor blinding, inadequate assessment or adjustment for other prognostic factors, and loss to follow-up. For RCTs, randomization and concealment procedures were often inadequately described. Summaries are provided in eFigures 1-4 in the Supplement.

### Studies Comparing Early vs Delayed BCG Vaccination in Infants Who Were Preterm or LBW

Ten studies compared BCG vaccination at or before 7 days with vaccination at later points in infants who were preterm or had LBW (Table). Gestational ages were between 31 to 36 weeks, except for the studies by Sedaghatian et al,^22,32^ which included neonates of 27 weeks’ gestational age and older (n = 181). Three studies^14,61,62^ included neonates who had LBW (<2.5 kg), and 1 study^20^ included neonates who weighed less than 2.4 kg, were full term, and were intrauterine growth restricted (IUGR).^14,20,61,62^ These studies are considered together, reflecting that accurate determination of gestational age is often not feasible; thus grouped consideration of infants who are preterm and/or had LBW is useful for health policy.

**Table.** Summary of Studies That Compared Infants Who Are Preterm or Have Low Birth Weights and Received BCG Vaccination Within 7 Days of Birth or at Later Points

Source	Country	Study Group	Study Type	First Group, No.	Additional Group(s), No.	Length of Follow-up	Outcomes	Key Results	Comments	Vaccine Strain
Biering-Sørensen et al,^62^ 2017	Guinea-Bissau	Neonates weighing 1.6 to 2.4 kg (10th-90th percentile) identified at maternity ward discharge or first contact with health center	Randomized clinical trial	2083 Received BCG “early”; exact days after birth not specified	2089 Received BCG at weight gain	Up to age 1 y	Mortalityfor reasons other than unintentional injury; enlarged lymph nodes	Early BCG was associated with a nonsignificant reduction in neonatal mortality (mortality rate ratio, 0.70 [95% CI, 0.47-1.04]) and infant mortality (0.88 [95% CI, 0.71-1.10]). At 6 mo, enlarged lymph nodes were found in 12 of 1416 infants (0.8%) in the early vaccination group and 7 of 1411 (0.5%) in the delayed group (*P* = .28)	Delayed group had 17% (n = 278/1631), 72% (n = 1179/1631), and 90% (n = 1226/1366) BCG vaccination coverage at 28 d, 2 mo, and 6 mo, respectively.	Statens Serum Institut Denmark 1331 for the early BCG group; control infants were mostly likely to receive BCG-Russia, provided by the local vaccination program.
Saroha et al,^21^ 2015	India	180 Preterm infants (31-33 weeks’ gestational age)	Randomized clinical trial	90 Received BCG within 72 h	90 Received BCG at 34 weeks’ post conception age	6 mo After vaccination	TST (5 TU of PPD), scar at 6 mo, IFN-γ in TST nonresponders, safety	No significant differences between groups on all outcomes. On follow-up, 2 of 69 infants (3%) vaccinated at 72 h and 2 of 48 (4%) vaccinated at 34 wk had nonsuppurative axillary lymphadenopathy	Large, unequal proportions did not complete the study owing to death or loss to follow-up (from 21 of 90 [23%] to 42 of 90 [47%]); IFN-γ levels were measured from unstimulated stored serum, not in response to PPD or BCG stimulation.	Danish 1331, prepared at Guindy, Chennai, India
Aaby et al,^61^ 2011 and Biering-Sørensen et al,^63^ 2012	Guinea-Bissau	2424 Well neonates weighing 0.69 to 2.5 kg, identified at maternity ward on discharge and 2 health centers	Randomized clinical trial	1218 Received BCG at birth	1206 Received BCG at weight gain or 2-mo vaccinations (median, 7.7 wk)	Up to age 1 y	Safety, all-cause mortality	No BCG-associated adverse outcomes. For group vaccinated at birth compared with those vaccinated at 2 mo/weight gain, mortality rate ratio at 1 mo, 0.52 (95% CI, 0.33-0.82); at 2 mo, 0.67 (95% CI, 0.47-0.95); at 6 mo, 0.75 (95% CI, 0.56-1.00); at 12 mo, 0.79 (95% CI, 0.61-1.02)	Gestational age not estimated; large losses to follow-up; HIV status not a predefined exclusion criterion	Statens Serum Institut Denmark 1331
Sedaghatian et al,^32^ 2009	United Arab Emirates	Preterm infants (27-36 weeks’ gestational age) and full-term neonates	Prospective cohort	52 Preterm infants and 31 infants who were full term and vaccinated at birth	29 Preterm infants vaccinated at full-term date	2 to 4 mo After vaccination	LTT, TST (10 TU of PPD), and scar	No significant differences in LTT, TST, and scar rates when preterm infants were vaccinated at birth vs near term dates	Very large losses to follow-up	Merieux seed derived from 1077 strain
Roth et al,^14^ 2004	Guinea-Bissau	Children in study area	Retrospective cohort	6 Children with scar data and 48 children with TST data who were born with LBW and vaccinated within 7 d of birth	36 Children with scar data and 103 children with TST data who were born with LBW and vaccinated later (age not specified)	To age 7.5 mo	TST, scar	Comparing infants who were LBW and vaccinated at age <7 d vs later: no significant differences; 3 of 6 in the first group were TST negative vs 15 of 36 in the second group (prevalence ratio, 1.2 [95% CI, 0.49-2.92]); 4 of 48 were scar negative in the first group vs 8 of 103 in the second group (1.07 [95% CI, 0.34-3.39]).	Different TST method used; reaction considered positive if diameter >2 mm. Infants with LBW of unknown gestational age were likely a combination of preterm, small for gestational age, and intrauterine growth restricted	Not stated
Thayyil-Sudhan et al,^23^ 1999	India	Preterm infants (<35 weeks’ gestational age)	Randomized clinical trial	31 Vaccinated at 34-35 weeks’ postconceptional age	31 Vaccinated at 38-40 weeks’ postconceptional age	6-8 wk after BCG vaccination	TST (1 TU of PPD), scar, LMIT, safety	No significant difference in the TST conversion rates (80% and 81%), positive LMIT (87% and 90%), or BCG scar (90% and 87%) between groups; no complications after vaccination.	Infants who were small for gestational age excluded	Danish 1331 manufactured at Guindy, Chennai, India
Sedaghatian et al,^22^ 1998	United Arab Emirates	Preterm infants (27-36 weeks’ gestational age) with AGA and infants who were full term with AGA; no exclusion for health status	Randomized clinical trial	70 Preterm infants vaccinated at birth	30 Preterm infants vaccinated at 40 weeks’ gestational age; 80 infants who were full term and vaccinated at birth	2-4 mo after BCG vaccination	TST (10 TU of PPD), scar	No differences in TST or BCG scars; birth weight associated with reactive TST in multivariable logistic regression model (odds ratio, 6.2 [95% CI, 1.9-20.6]). Subgroup analysis of neonates born at 27 to 33 weeks’ gestational age vaccinated at birth (n = 14) or term (n = 13): no significant difference in scar size (1.7 vs 3.0 mm; mean difference, 1.3 [95% CI, −1.3 to 3.9] mm) or TST induration (3.4 vs 4.2 mm; mean difference, 0.8 [95% CI, −2.3 to 3.9] mm)	Very large losses to follow-up (34 of 70 [49%] in the group vaccinated at birth and 14 of 30 [47%] in the group vaccinated late)	Lyophilized BCG, Behring Laboratories, Germany
Mussi-Pinhata et al,^20^ 1993	Brazil	Infants who were full term with symmetric intrauterine growth restriction (<5th centile, 1710-2400 g; median, 2170 g); further AGA group	Randomized clinical trial	16 Vaccinated within 5 d	17 Children at gain to 2500 g; 16 at 3 mo; 6 at 6 mo.	12-14 wk after BCG vaccination	LTT, TST (5 TU of PPD), and scar, safety	No differences for LTT or TST between BCG vaccination at birth vs later points; all had BCGs scar at 1-2 mo; none had adverse reactions up to 3 mo post vaccination	3-Mo point used for delayed vaccination group in meta-analysis	Moreau, Rio de Janeiro
Dawodu,^31^ 1985	Nigeria	Preterm infants with AGA (32-36 weeks’ gestational age) and infantswho were full term with AGA	Nonrandomized clinical trial	12 Preterm infants vaccinated at birth	15 Infants who were full term vaccinated at birth; 8 preterm infants vaccinated at estimated due date	2 mo after BCG vaccination for TST, 6 months after BCG vaccination for safety	TST (10 TU of PPD), scar, safety	TST conversion rates (10/12 [83%], 14/15 [93%], and 7/8 [88%]) and BCG scar rates (11/12 [92%], 14/15 [93%], and 7/8 [88%]) did not differ significantly; no unusual reactions to vaccination on 6-mo follow-up.	TST done when still very young	Not stated

Abbreviations: AGA, appropriate for gestational age; BCG, bacillus Calmette-Guérin; IFN-γ, interferon gamma; LTT, lymphocyte transformation test; PPD, purified protein derivative; TST, tuberculin skin test; TU, tuberculin units.

Seven studies^14,20,21,22,23,31,32^ reported scar formation and TST conversion and were therefore included in a meta-analysis. Only data on infants who were preterm and/or LBW and had follow-up adequate to record scar formation (n = 515) or TST conversion (n = 397) were included.

### Safety, Reactogenicity, and Mortality

Six studies evaluated safety. A large RCT from Guinea-Bissau reported enlarged lymph nodes at 6 months in 12 of 1416 infants (0.8%) with LBW who were vaccinated early and 7 of 1411 infants (0.5%) with LBW who were vaccinated at later points (reported *P* = .28).^62^ A trial in India on 180 neonates born between 31 to 33 weeks’ gestational age reported 2 infants in each of the early and late vaccination groups with nonsuppurative lymphadenopathy (a total of 4 individuals).^21^ There were no other adverse reactions, and meta-analysis was not possible.

**Figure 2.**
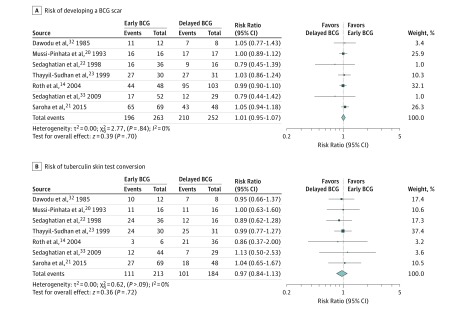
Meta-analyses A, Meta-analysis of the risk of developing a bacillus Calmette-Guérin (BCG) scar. B, Meta-analysis of tuberculin skin test conversion in infants who are preterm and/or had low birth weights for BCG vaccination within 7 days or at a later point.

The formation of BCG scars (defined by study authors) was reported in 7 studies, with no significant difference between early and delayed vaccination in meta-analysis (Figure 2A). In 3 RCTs of 6492 infants who had LBW in Guinea-Bissau, early BCG vaccination reduced all-cause neonatal mortality (reported mortality rate ratios by meta-analysis, 0.62 [95% CI, 0.46-0.83]) and infant mortality (0.84 [95% CI, 0.71-1.00]).^61,62,63^

### Immunogenicity

Two studies reported lymphocyte transformation test (LTT) results,^20,32^ and 1 study reported lymphocyte migration inhibition test (LMIT) results^23^ without differences between early and later vaccination. One study comparing infants who were moderately preterm and vaccinated within 7 days of birth or at 34 weeks’ postconception age reported no difference at age 6 months in interferon-γ levels in the unstimulated serum of infants who had not responded to the TST.^21^

### TST Conversion

All 7 studies of TST conversion (with thresholds defined by study authors) reported no significant difference between early and delayed BCG vaccination.^14,20,21,22,23,31,32^ The pooled estimate in meta-analysis also showed no difference and low heterogeneity between studies (Figure 2B).

We performed post hoc sensitivity analyses for both BCG scar formation and TST conversion by assessing changes in pooled effect size after exclusion of 1 study of neonates who were full term and IUGR,^20^ the only retrospective study,^14^ and 1 study where delayed vaccination was performed relatively early, at 34 weeks postconception.^21^ We found no significant changes in the pooled estimate or estimated heterogeneity after these exclusions. Funnel plots of included studies showed no indication of nonsymmetry, suggesting minimal reporting bias (eFigure 5 in the Supplement).

### Efficacy

No studies reported efficacy of BCG against TB disease. In an RCT^61^ of 2424 infants, there were no deaths in infants who had LBW and were exposed to TB in either the early or delayed BCG arms.

### Descriptive Studies of Early or Delayed BCG Vaccination of Infants Who Are Preterm and/or LBW

Further data on safety were extracted from descriptive (noncomparative) studies (eTable 1 in the Supplement) and studies making comparisons between infants who were preterm and/or had LBW and those who were full term and/or had NBWs (eTable 2 in the Supplement). A Danish RCT comparing BCG vaccination within 7 days in 71 infants who were preterm (>32 weeks’ gestational age) and 61 infants who had LBW (1.0 to 2.5 kg) vs infants who received no BCG vaccination reported no difference in all-cause hospitalization or psychomotor development at age 22 months.^65,66^ A larger analysis of 1633 neonates who weighed less than 2.5 kg at birth in the arm receiving early BCG vaccination in an RCT in Guinea-Bissau reported a frequency of 3 of 1110 infants (0.3%) with enlarged lymph nodes 6 months after vaccination.^34^ Additionally, the authors reported that 765 of 1097 infants (70%) were hospitalized or sought a consultation at the hospital during the first 6 months of life. It was not possible to ascertain if this was typical in the population or the association with BCG vaccination. No vaccine-associated complications in infants who were preterm were reported in another study of early BCG vaccination^38^ and 2 studies of delayed vaccination.^39,40^

### Studies Comparing infants Who Are Preterm and/or had LBW With Infants Who Are Full-Term and/or had NBWs

**Figure 3.**
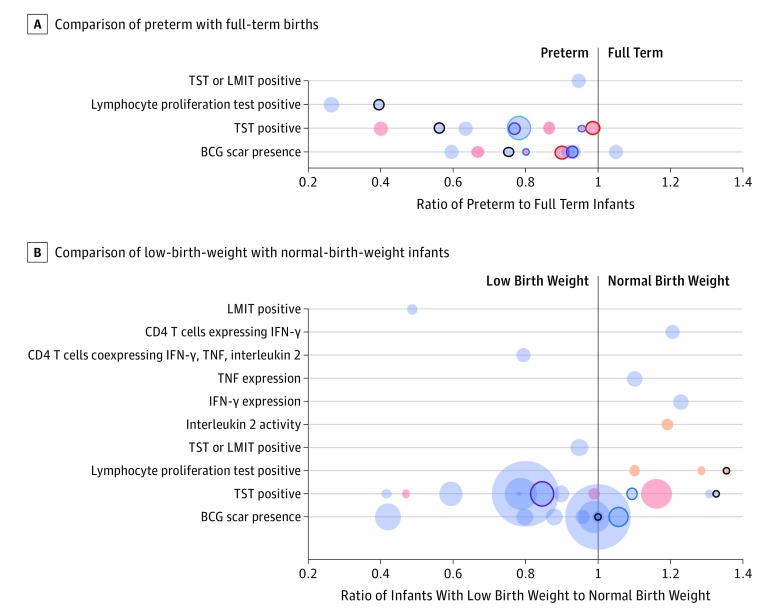
Responses to BCG Vaccination From Included Studies A and B, Comparisons of vaccination responses in infants who are preterm vs full-term and comparisons of vaccination responses in infants who have low birth weight vs normal birth weight. Each circle represents the ratio of responses for a specific outcome from a single study. The size of each circle is proportional to the number of infants who were preterm and/or had low birth weights. No estimate of variability is given, and pooling of results was not justified. Ratios of mean diameter (pink circles) or reported adjusted odds ratios were used in preference to unadjusted ratios of proportions (purple circles), where available. Mean stimulation index outcomes are represented by orange circles. Circles with a solid outline represent responses from studies that included vaccination after 7 days postbirth of infants who were preterm and/or had low birth weights (eTable 2 in the Supplement). BCG indicates bacillus Calmette-Guérin; IFN-γ, interferon γ; LMIT, leukocyte migration inhibition test; TNF, tumor necrosis factor; TST, tuberculin skin test.

Six studies^41,44,45,48,49,58^ evaluated safety, with 4 reporting no vaccine-associated complications in infants who are preterm and/or LBW receiving early or delayed BCG vaccination.The RCT^48^ in South Africa included 2419 neonates who had LBW and 2019 who were preterm who had all received early BCG vaccination; this study included detailed reporting of adverse reactions. Individual patient data were obtained and post hoc comparisons made with 8299 infants who were full term and/or had NBWs and received early BCG vaccination. Across all trial participants, 22 grade 3 and 4 vaccine-associated adverse reactions occurred, including keloid scars (n = 16), suppurative lymphadenitis (n = 5), and disseminated BCG disease (n = 1). The relative risk of suppurative lymphadenitis in infants who had LBW (0.12%) compared with infants who had NBWs (0.02%) was sizeable, but low frequency and a lack of significance (relative risk, 5.7 [95% CI, 0.95-34]), as well as a lack of adjustment for the likely confounding factor of HIV status, which was not systematically assessed at birth, compromised the findings. There were no differences in other safety outcomes or episodes of suppurative lymphadenitis in infants who were preterm. None of the 186 deaths were considered by investigators to be associated with BCG vaccination. A 1955 study by Gaisford^45^ reported an increased risk of adenitis in infants who were preterm, although quantitative data were not presented. Several studies compared BCG scar formation between infants who were preterm and/or had LBW and those who were full term and/or had NBWs, with an indication of less reactogenicity particularly in the preterm group (Figure 3).

Reported measures of immunogenicity (lymphocyte proliferation and migration tests, cytokine responses, including tumor necrosis factor, interferon-γ, and interleukin 2) were more frequently studied in infants who had LBW, with no clear differences compared with those who had NBWs. Most studies comparing TST conversion between infants who were preterm and full term reported reduced responses in the preterm group, although the data were not suitable for meta-analysis (Figure 3).

The use of different outcomes and summary statistics in each study, considerable heterogeneity in study design, and risks of bias in these predominantly observational studies meant that they were not suitable for pooled or meta-analysis. The differences in findings between studies did not suggest a pattern based on BCG vaccine strain.

### Efficacy

Three studies included infants who were preterm and/or had LBW and were vaccinated early and reported TB disease outcomes.^45,48,57^ Gaisford et al^45^ reported no cases of TB in a cohort of 5215 vaccinated newborns in the United Kingdom during 4 years of follow-up between 1949 and 1954, compared with 13 cases among children who did not receive vaccinations. Hawkridge et al^48^ included infants who were preterm and had LBW in a South African RCT comparing intradermal with percutaneous BCG vaccinations. Individual patient data were obtained; however, because data on prognostic variables affecting TB outcome, such as HIV status and maternal TB, were only collected in infants subsequently investigated on suspicion of TB disease, we were unable to perform a meaningful post hoc, cross-arm analysis. Furthermore, in the absence of a study arm that included infants who were preterm and/or had LBW who received delayed or no BCG vaccinations, no conclusions could be drawn in this respect. A case-control study of 130 BCG-vaccinated children up to 15 years of age in Thailand compared children with TB with matched controls. Age-adjusted univariate analysis reported by the authors did not identify LBW as a risk factor for TB, but the study was likely underpowered for this exposure.^57^ An observational study in Guinea-Bissau including infants who had LBW in 2 cohorts that received BCG vaccination at a median age of 9 days found that LBW was not an independent risk factor for all-cause 12-month mortality. However, only about half of all deaths and just over half of survivors had birth weight data.^46^

## Discussion

We report the first systematic review of BCG safety, immunogenicity, and protective efficacy in infants who are preterm and/or LBW. There was no evidence of increased BCG-associated adverse events when vaccination was administered within 7 days of birth in clinically stable neonates, compared with vaccination at later points. Meta-analyses of BCG scar formation and TST conversion suggested no differences between the 2 groups. Immunogenicity could not be evaluated owing to inadequate data. No studies reported efficacy against TB disease in BCG within 7 days vs vaccination at later points. Studies of adverse reactions comparing infants who were preterm and/or had LBW to infants who were full term and/or had NBWs predominantly reported no differences.

### Strengths

There were a large number of infants who were preterm and/or had LBW (n = 7281) vaccinated early or late, informing conclusions on safety. In most studies, reactogenicity was measured using BCG scar formation or TST, allowing cross-comparison and greater confidence in observed effects.

Across studies, neonates with signs of severe illness were excluded. Where defined, common criteria for exclusion were neonatal sepsis, fever, assisted ventilation, hemodynamic instability, congenital malformations, asphyxia, maternal serious illness, TB, known HIV, or hepatitis B. Only 7 studies clearly included neonates younger than 30 weeks’ gestational age or less than 1.5 kg birth weight. This is unsurprising, as most extremely neonates who are preterm and/or had very LBW may be clinically unstable in the first few days of life. Furthermore, neonates born at more than 30 weeks’ gestational age and weighing more than 1.5 kg constitute the largest portion of the overall cohort of infants who are preterm and/or had LBW, justifying a focus on this cohort globally.

### Safety

Four studies, 3 of which had large losses to follow-up, reported all-cause mortality. None showed increased mortality among infants who had LBW and received early BCG vaccination, compared with either (1) vaccination at later points in infants who had LBW or (2) BCG vaccination within 7 days in infants who had NBW.^46,61,62,63^ Subgroup analysis of raw data from the RCT by Hawkridge et al^48^ showed a nonsignificant increase in lymphadenitis in infants who had LBW but was unadjusted for the major confounder of maternal HIV, which is associated with preterm birth, LBW, and TB risk.^67^ The overall incidence of lymphadenitis was low (0.12% in infants who had LBW) and is unlikely to be of clinical importance in the context of current WHO advice. There was no increase in lymphadenitis in infants who were preterm, but most studies reported lower markers of reactogenicity in the form of BCG scar formation compared with infants who were full term (Figure 3).

No cases of disseminated BCG were reported in infants who are preterm and/or LBW. In comparison, Hesseling et al^18^ reported a 1% incidence of disseminated BCG disease in HIV-infected infants. There is currently uncertainty regarding the most appropriate timing for BCG vaccination in HIV-exposed or HIV-infected children, and studies are ongoing.^68,69^ Serious adverse events in immunocompetent neonates receiving BCG vaccination are uncommon; the WHO estimates 1 severe local adverse event per 1000 to 10 000 doses. Systemic adverse events such as disseminated BCG disease are rare, estimated at 1 case per 230 000 to 640 000 doses.^19^ A much larger number of infants who are preterm and/or had LBW is needed to detect an increased incidence of severe adverse events.

### Immunogenicity and TST Conversion

Few studies evaluated immunogenicity by antigen-specific T-cell cytokine responses to PPD, BCG or *Mycobacterium tuberculosis* antigens. Tuberculin skin test was the most frequently reported marker of vaccine response. The traditional view is that TST conversion reflects the development of delayed-type hypersensitivity (DTH) to mycobacterial antigens and thus vaccine take.^70^ While this view has informed current BCG vaccination policy, its validity remains uncertain, because correlates of protection remain poorly defined.^26,70,71^ The rate of TST conversion is dependent on host-associated factors (eg, age, dose of vaccine, and time after vaccination) and test-associated factors (eg, technique and strength of tuberculin), as well as environmental mycobacteria.^72^ Furthermore, TST may be falsely negative in early life and malnutrition.^72,73^ This may be particularly relevant, because none of the studies corrected for gestational age, and infants who are IUGR may be biologically similar to malnourished children. All studies performed TST at chronological ages of 6 months or less. It is possible that infants who are preterm and/or had LBW who show poor responses to a heterogeneously applied TST may still develop useful immunity to severe forms of TB disease. The use of BCG scar formation and TST response at least allows meta-analysis of a standardized measure of delayed-type hypersensitivity. Mindful of the above limitations, our findings suggest consistently similar delayed-type hypersensitivity in infants who are preterm and/or had LBW that received BCG vaccination within 7 days or at a later point.

### Limitations

In all studies, gestational age was estimated using Ballard or Dubowitz scores and may be inaccurate. Most RCTs had poorly described randomization, allocation concealment methods, and blinding. Many studies were unblinded. This may be of relevance because TST is known to be operator dependent, but it is difficult to gauge the direction of this potential bias.

The TSTs were performed at varying times after BCG vaccination using varying methods. Assessment of confounders of TST reaction, such as malnutrition, HIV, and concurrent TB infection, were scarcely reported.^72^ While there appears to be a high degree of consistency in the outcomes for the studies included in the meta-analysis, these factors may have contributed to the significant variation in TST and BCG scar outcomes across all studies.

Few studies actively monitored for adverse reactions. Notably, Hawkridge et al^48^ conducted 3 months of active follow-up of 4851 infants for adverse reactions. However, many studies suffered from large losses to follow-up, and the risk of underestimation of adverse reactions is significant. Different BCG strains were used between studies, possibly giving variable reactogenicity.^74^

Cohort studies comparing BCG responses in infants who were preterm vs those who were full term suggested a direction of effect of weaker BCG scar formation, TST conversion, and lymphocyte proliferation in infants who were preterm, which could not be confirmed with meta-analysis. We would caution against drawing conclusions based on these data. Importantly, there is no evidence that infants who are preterm would respond better if vaccination occurs more than 7 days after birth, because 3 of the 5 studies comparing delayed BCG vaccination in infants who are preterm vs full term showed less BCG scar formation, TST conversion, and lower lymphocyte proliferation test responses in infants who were preterm.^22,32,41,42,51^

### Conclusions and Policy Implications

Most studies included healthy neonates born at more than 30 weeks’ gestational age or weighing more than 1.5 kg and reported no increased risk of adverse reactions or infant mortality after BCG vaccination within 7 days of birth compared with BCG vaccination at later points. Vaccination within 7 days resulted in similar rates of BCG scar formation and TST conversion compared with later vaccination. No studies evaluated protective efficacy comparing early vs late BCG. Assessment of TB disease outcomes would require large RCTs in areas with high TB burden. Future research should focus on addressing the highlighted methodological weaknesses, measuring specific immune responses, including antigen-specific intracellular cytokine responses to purified protein derivative or *Mycobacterium tuberculosis* antigens.^25^

Our independent findings strongly support the most recent WHO advice of early vaccination in healthy infants born moderately preterm and with moderate LBW, and synthesizes more extensively the evidence for early vaccination safety in this population.^11^ The question of when best to vaccinate these neonates at the local level requires consideration of this evidence, the potential outcomes of reduced vaccine coverage if BCG vaccination is delayed, service delivery factors, and the results of further trials on nonspecific outcomes of early BCG vaccination. At present, on the basis of current evidence, a uniform policy of early BCG vaccination of clinically stable infants who are preterm and/or had LBW appears justified.

## Notes

Supplement. **eMethods.** Search Strategy **eFigure 1.** Risk of Bias Summary: Review Authors' Judgements About Each Risk of Bias Item for Randomized Trials. **eFigure 2.** Risk of Bias Summary: Review Authors' Judgements About Each Risk of Bias Item for Cohort Studies in Table 1. **eFigure 3.** Risk of Bias Summary: Review Authors' Judgements About Each Risk of Bias Item for Cohort Studies in eTable 1. **eFigure 4.** Risk of Bias Summary: Review Authors' Judgements About Each Risk of Bias Item for Cohort Studies in eTable 2. **eFigure 5.** Funnel Plots for Studies Included in the Meta-analyses **eTable 1.** Summary of Studies That Compared Preterm or Low-Birthweight Infants Receiving Either Early or Delayed BCG to Unvaccinated Controls or No Control Group **eTable 2.** Summary of Studies Comparing BCG in Preterm/Low-Birthweight Infants to Full-term/Normal-birthweight Infants.
