# Supplementary material for: Safety and Immunogenicity of Early Bacillus Calmette-Guérin Vaccination in Infants Who Are Preterm and/or Have Low Birth Weights: A Systematic Review and Meta-analysis
Source: JAMA Pediatr. 2018 Nov 26;173(1):75–85. doi: 10.1001/jamapediatrics.2018.4038 (PMC6583455; doi:10.1001/jamapediatrics.2018.4038)
Supplement: Supplement. — eMethods. Search Strategy eFigure 1. Risk of Bias Summary: Review Authors' Judgements About Each Risk of Bias Item for Randomized Trials. eFigure 2. Risk of Bias Summary: Review Authors' Judgements About Each Risk of Bias Item for Cohort Studies in Table 1. eFigure 3. Risk of Bias Summary: Review Authors' Judgements About Each Risk of Bias Item for Cohort Studies in eTable 1. eFigure 4. Risk of Bias Summary: Review Authors' Judgements About Each Risk of Bias Item for Cohort Studies in eTable 2. eFigure 5. Funnel Plots for Studies Included in the Meta-analyses eTable 1. Summary of Studies That Compared Preterm or Low-Birthweight Infants Receiving Either Early or Delayed BCG to Unvaccinated Controls or No Control Group eTable 2. Summary of Studies Comparing BCG in Preterm/Low-Birthweight Infants to Full-term/Normal-birthweight Infants. [file jamapediatr-173-75-s001.pdf]

Supplementary Online Content

Badurdeen S, Marshall A, Daish H, Hatherill M, Berkley JA. Safety and immunogenicity of early Bacillus Calmette-Guérin vaccination in infants who are preterm and/or have low birth weights: a systematic review and meta-analysis. *JAMA Pediatr.* Published online November 26, 2018.
doi:10.1001/jamapediatrics.2018.4038

eMethods. Search Strategy
eFigure 1. Risk of Bias Summary: Review Authors' Judgements About Each Risk of Bias Item for Randomized Trials.
eFigure 2. Risk of Bias Summary: Review Authors' Judgements About Each Risk of Bias Item for Cohort Studies in Table 1.
eFigure 3. Risk of Bias Summary: Review Authors' Judgements About Each Risk of Bias Item for Cohort Studies in Supplement Table 1.
eFigure 4. Risk of Bias Summary: Review Authors' Judgements About Each Risk of Bias Item for Cohort Studies in Supplement Table 2.
eFigure 5. Funnel Plots for Studies Included in the Meta-analyses
eTable 1. Summary of Studies That Compared Preterm or Low-Birthweight Infants Receiving Either Early or Delayed BCG to Unvaccinated Controls or No Control Group.
eTable 2. Summary of Studies Comparing BCG in Preterm/Low-Birthweight Infants to Full-term/Normal-birthweight Infants.

This supplementary material has been provided by the authors to give readers additional information about their work.

© 2018 Badurdeen S et al. *JAMA Pediatrics.*

eMethods. Search Strategy

All searches performed in Ovid SP.

Medline:

1. exp BCG Vaccine/ or 'bcg'.mp. or (bacill$ adj3 Calmette$).mp. or Calmette Vaccin$.mp.
2. exp Infant, Low Birth Weight/ or exp Infant, Premature/ or "Birth Weight".mp. or "Low birth weight".mp. or "Very Low birth weight".mp. or "Extremely Low birth weight".mp. or "Growth Retardation".mp. or "Premat*".mp. or "Preterm".mp. or "Small for Gestational Age".mp.
3. 1 and 2

Embase:

1. exp BCG Vaccine/ or 'bcg'.mp. or (bacill$ adj3 Calmette$).mp. or Calmette Vaccin$.mp
2. exp prematurity/ or exp low birth weight/ or exp small for date infant/ or "Birth Weight".mp. or "Low birth weight".mp. or "Very Low birth weight".mp. or "Extremely Low birth weight".mp. or "Growth Retardation".mp. or "Premat*".mp. or "Preterm".mp. or "Small for Gestational Age".mp.
3. 1 and 2

Global Health:

1. exp BCG Vaccine/ or 'bcg'.mp. or (bacill$ adj3 Calmette$).mp. or Calmette Vaccin$.mp.
2. exp premature infants/ or exp low birth weight infants/ or "Birth Weight".mp. or "Low birth weight".mp. or "Very Low birth weight".mp. or "Extremely Low birth weight".mp. or "Growth Retardation".mp. or "Premat*".mp. or "Preterm".mp. or "Small for Gestational Age".mp.
3. 1 and 2

Randomized trials search (filter from Cochrane Highly Sensitive Search Strategy for identifying randomized trials in Medline- Cochrane Handbook):

Medline:

1. exp infant/ or infants or exp newborn/ or newborn*.mp or newborn.mp or 'new born'.mp or exp Child/ or child.mp or children.mp or baby.mp or babies.mp or neonat*.mp
2. exp BCG Vaccine/ or 'bcg' or (bacill$ adj3 Calmette$) or Calmette Vaccin$
3. 1 and 2
4. randomized controlled trial.pt.
5. controlled clinical trial.pt.
6. randomized.ab.
7. placebo.ab.
8. clinical trials as topic.sh.
9. randomly.ab.
10. trial.ti.
11. 4 or 5 or 6 or 7 or 8 or 9 or 10
12. exp animals/ not humans.sh.
13. 11 not 12
14. 3 and 13

© 2018 Badurdeen S et al. *JAMA Pediatrics.*

Global Health:

1. exp infants/ or infants.mp. or exp newborn/ or newborn*.mp. or 'newborn'.mp. or 'new born'.mp. or child.mp. or children.mp. or baby.mp. or babies.mp. or neonat*.mp.
2. exp BCG Vaccine/ or 'bcg' or (bacill$ adj3 Calmette$) or Calmette Vaccin$
3. exp clinical trials/ or exp randomized controlled trials/ or trial.mp. or trial*
4. 1 and 2 and 3

Embase:

1. exp infant/ or infants.mp. or exp newborn/ or newborn*.mp. or 'newborn'.mp. or 'new born'.mp. or 'child'.mp. or 'children'.mp. or 'baby'.mp. or 'babies'.mp. or neonat*.mp.
2. exp BCG Vaccine/ or 'bcg'.mp. or (bacill$ adj3 Calmette$).mp. or Calmette Vaccin$.mp
3. exp randomized controlled trial/ or exp crossover procedure/ or exp double-blind procedure/or exp single-blind procedure/
4. (((((random$ or factorial$ or crossover$ or cross over$ or cross-over$ or placebo$ or doubl$) adj blind$) or singl$) adj blind$) or assign$ or allocat$ or volunteer$).mp.
5. 3 or 4
6. 1 and 2 and 5

© 2018 Badurdeen S et al. *JAMA Pediatrics.*

eFigure 1. Risk of Bias Summary: Review Authors' Judgements About Each Risk of Bias Item for Randomized Trials. Studies in Table 1 are Indicated with an *.

	Random sequence generation (selection bias)	Allocation concealment (selection bias)	Blinding of participants and personnel (performance bias)	Blinding of outcome assessment (detection bias)	Incomplete outcome data (attrition bias)	Selective reporting (reporting bias)	Other bias
Aaby et al. 2011 & Biering-Sorensen et al. 2012*	+	+	+	−	−	+	+
Biering-Sorensen et al. 2017*	+	−	−	+	+	+	−
Mussi-Pinhata et al. 1993*	−	−	+	+	+	+	+
Saroha et al 2015*	+	+	+	−	−	+	−
Sedaghatian et al. 1998*	−	−	+	−	−	+	−
Thayyil-Sudhan et al. 1999*	−	−	+	−	+	+	−
Kjaergaard et al. 2016	+	+	−	+	−	+	+
Stensballe et al. 2017	+	+	−	+	+	+	+

© 2018 Badurdeen S et al. *JAMA Pediatrics.*

eFigure 2. Risk of Bias Summary: Review Authors' Judgements About Each Risk of Bias Item for Cohort Studies in Table 1.

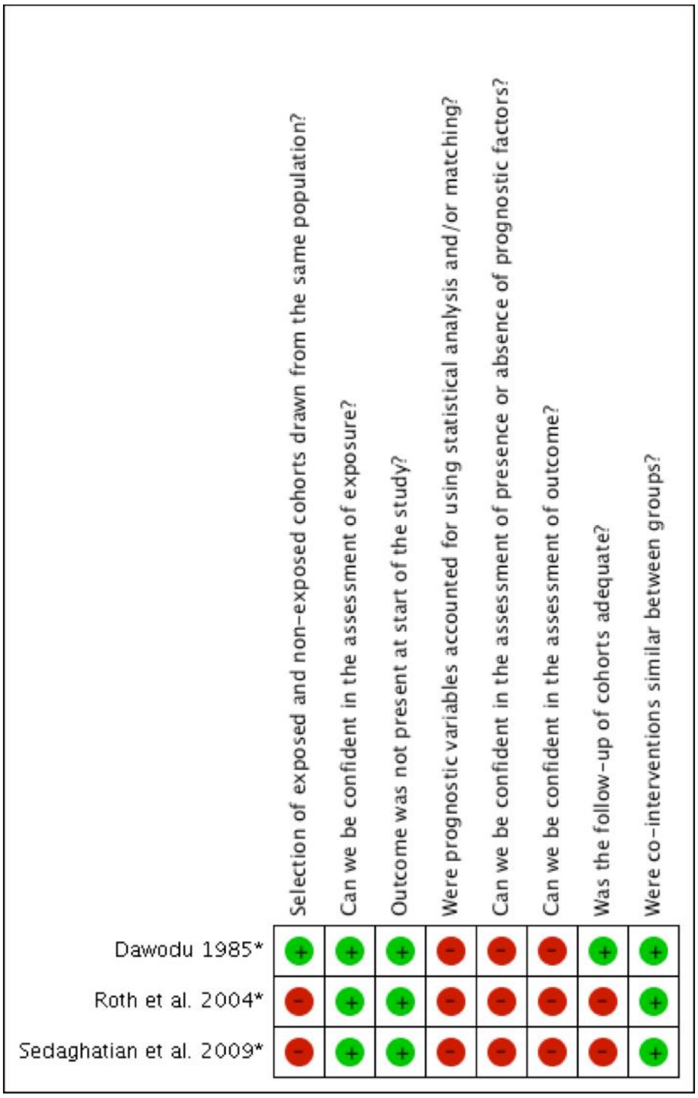

© 2018 Badurdeen S et al. *JAMA Pediatrics.*

eFigure 3. Risk of Bias Summary: Review Authors' Judgements About Each Risk of Bias Item for Cohort Studies in Supplement Table 1.

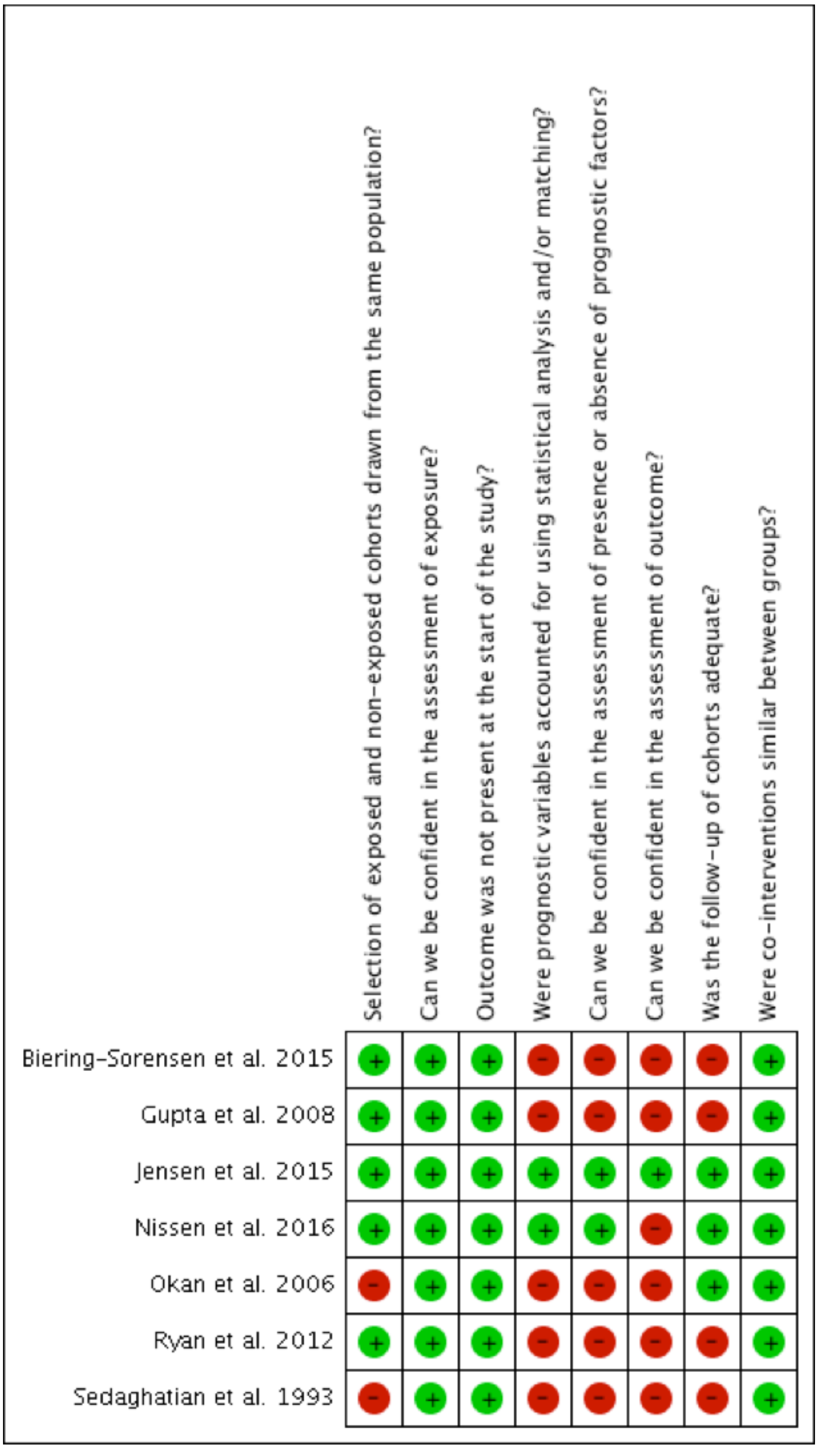

© 2018 Badurdeen S et al. *JAMA Pediatrics.*

eFigure 4. Risk of Bias Summary: Review Authors' Judgements About Each Risk of Bias Item for Cohort Studies in Supplement Table 2.

	Selection of exposed and non–exposed cohorts drawn from the same population?	Can we be confident in the assessment of exposure?	Outcome was not present at the start of the study?	Were prognostic variables accounted for using statistical analysis and/or matching?	Can we be confident in the assessment of presence or absence of prognostic factors?	Can we be confident in the assessment of outcome?	Was the follow–up of cohorts adequate?	Were co–interventions similar between groups?
Camargos et al. 2006	+	+	+	−	−	+	−	+
Cebeci et al. 2017	−	−	+	−	−	−	+	+
Faridi et al. 2009	−	+	+	−	−	−	+	+
Ferreira et al. 1996	+	+	+	−	−	−	−	+
Gaisford 1955	+	+	+	−	−	+	−	−
Garly et al. 2003	+	+	+	+	−	+	−	−
Grindulis et al 1984	+	+	+	−	−	+	+	+
Hawkridge et al. 2008	+	+	+	−	−	+	+	+
Kaur et al. 2002	+	+	+	−	−	−	+	+
Manerikar et al. 1976	−	+	+	−	−	−	+	+
Negrete–Esqueda et al. 2007	+	−	+	−	−	−	+	+
Neumann et al. 1998	−	−	+	−	−	−	+	+
Osendarp et al. 2006	+	+	+	−	−	−	+	+
Roth et al. 2005	−	−	+	−	−	−	−	+
Sartono et al. 2010	+	+	+	−	−	−	−	+
Sepulveda et al. 1994	+	+	+	−	−	+	+	+
Timmermann et al. 2015	−	+	+	+	+	−	−	+
Tipaya–mong–khogul et al. 2005	+	+	+	−	−	+	+	+
Tun et al. 2000	−	+	+	−	−	−	+	+
Verma et al. 1995	−	+	+	−	−	−	−	+

© 2018 Badurdeen S et al. *JAMA Pediatrics.*

eFigure 5. Funnel Plots for Studies Included in the Meta-analyses

1. BCG scar

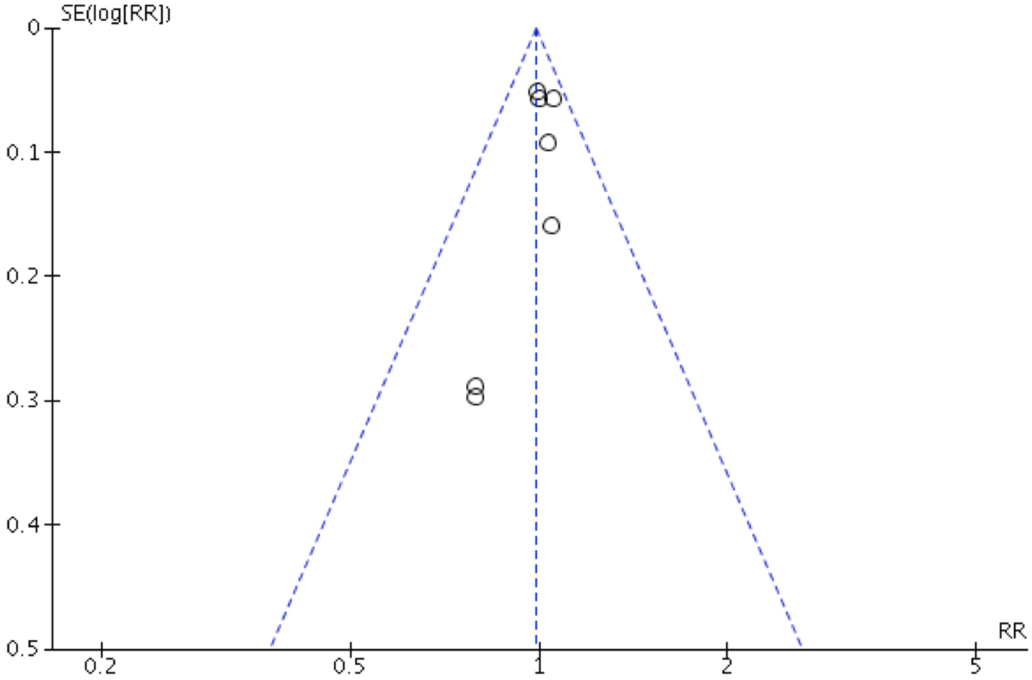

2. TST positivity

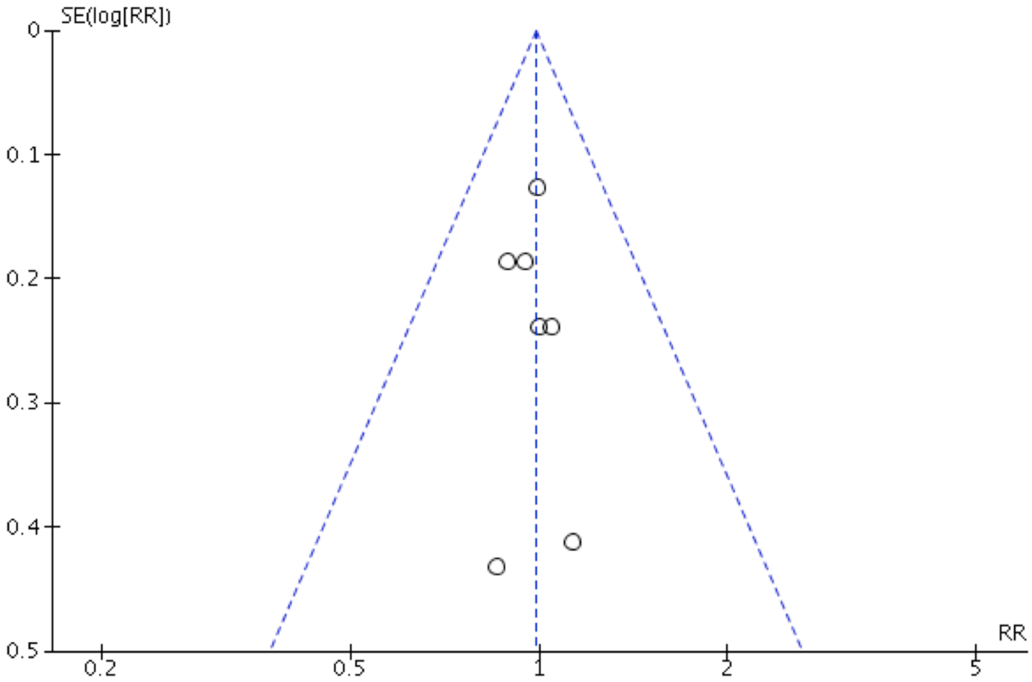

© 2018 Badurdeen S et al. *JAMA Pediatrics.*

eTable 1. **Summary of Studies That Compared Preterm or Low-Birthweight Infants Receiving Either Early or Delayed BCG to Unvaccinated Controls or No Control Group**

Citation	Study group	Study type	Outcome	Key Result	Comments
Stensballe et al,[66] 2017; Nissen et al,[38] 2016 & Kjaergaard et al,[67] 2016<br><br>Denmark	Newborns (n=4262) were randomized to early BCG or no BCG. Inclusion criteria were > 32 gw and birthweight > 1000 g<br><br>Trial included 144 neonates <37 gw, (of which 71 were allocated to BCG) and 123 LBW neonates <2.5kg (of which 61 were allocated to BCG).<br><br>Follow-up until 15 months of age.	Randomized controlled trial	All-cause hospitalisation, safety<br><br>Psychomotor development at 22 months age	No significant differences between trial arms in either PT or LBW subgroups.<br><br>Overall incidences of regional and suppurative lymphadenitis were 6.1/1000 and 4.7/1000 respectively. No subgroup data on PT/LBW infants. No BCG related deaths were reported.<br><br>No differences in Ages and Stages Questionnaire scores between PT infants that did and did not receive BCG	Strain- Danish BCG 1331
Biering-Sørensen et al,[35] 2015<br><br>Guinea-Bissau	1633 neonates <2.5 kg were randomized to BCG at birth and examined for scar at 12 months; a subgroup was tested for PPD response at 2 and 6 months.	Nested prospective cohort	TST, scar<br><br>Safety (peak incidences during follow-up shown)	TST positive in 28-44%.<br>Scar present in 92-99%.<br><br>“Abscess or scab”<br>2 months: 305/1274 (24%)<br><br>“Enlarged lymph node”<br>2 months: 4/1277 (0.3%)<br>6 months: 3/1110 (0.3%)<br>“Fever”<br>3 days: 76/1323 (5.7%)<br><br>“Hospitalization/ consultations”<br>6 months: 765/1097 (70%)	A lower cut-off of 1 mm for TST positive response was used.<br>TST and scar response was influenced by slow vs normal growth subtypes of BCG vaccine used (Danish 1331 strain).<br>Unable to differentiate between the respective incidences of abscess and scab, or to ascertain how many cases of “enlarged lymph node” were complicated, for example by lymphadenitis

© 2018 Badurdeen S et al. *JAMA Pediatrics.*

Citation	Study group	Study type	Outcome	Key Result	Comments
Jensen et al,[37] 2015<br><br>Guinea-Bissau	260 infants <2.5 kg vaccinated at birth, compared to 207 unvaccinated infants. Cytokine responses assessed at 4 weeks (±7 days)	Nested prospective cohort	In-vitro cytokine responses to PPD (positive control experiment)	For all cytokines, BCG vaccination was associated with a significantly higher response to PPD.<br>(IL-1β, IL-5, IL-6, IL-10, IL-17, IFN-γ and TNF-α)	Strain- SSI Denmark
Ryan et al,[40] 2012 Ireland (abstract)	35 babies > 34 gw and < 2.5 kg immunized at discharge. The mean age at immunization was 9.6 days and mean weight was 2190 g	Prospective cohort	Safety	No immediate complications related to the vaccine were noted.	BCG strain not stated
Gupta et al,[36] 2008<br><br>India	102 twin pairs ≥33 gw and birth weight ≥1500 g were vaccinated within 48 h of birth. Follow up at 14 weeks.	Prospective cohort	Local reaction	Local reaction was seen in 84% babies and scar formed in 41%.	Danish 1331 strain BCG Laboratory, Guindy, Chennai
Okan et al,[39] 2006<br><br>Turkey	35 infants with mean gestational age of 32.4 weeks (range 28–35) were included. At the time of vaccination, the mean post-menstrual age was 44.1 weeks (range 37-45), and the mean body weight was 3888 g.<br><br>TSTs were conducted 8–16 weeks after the vaccination	Prospective cohort	TST, scar, safety	20 (57%) infants had a positive TST response. 34 babies (97%) had a BCG scar. The mean body weight of the TST positive babies was significantly higher than TST negative babies at the time of vaccination (P=0.042)<br><br>No complications were observed.	Suppurative adenitis, skin ulceration and abscess formation were specifically monitored.<br><br>Strain- Serum Institute of India, Hadapsar, India
Sedaghatian et al,[41] 1993<br><br>UAE	289 PT infants (26-37 weeks) received BCG at birth. 101 infants were evaluated 2-4 months after vaccination. Unwell infants requiring inpatient care were not excluded.	Prospective cohort	TST, scar, safety	32% had TST >5mm<br><br>68% had a BCG scar<br><br>No vaccine complications were observed.	Very large losses to follow up. TST done when still very young.<br><br>Strain- lyophilized BCG, Behring laboratories, Germany

Abbreviations: AGA, appropriate for gestational age; aOR, adjusted odds ratio; CI, confidence interval; FT, full term; GA, gestational age; gw, gestational weeks; IUGR, intrauterine growth retardation; LBW, low birthweight; LMIT, lymphocyte migration inhibition test; LTT, lymphocyte transformation test; NBW, normal birthweight; PT, preterm; RCT, randomized controlled trial; SGA, Small for Gestational Age; SSI, Statens Serum Institut; TST, tuberculin skin test; TU, tuberculin units

© 2018 Badurdeen S et al. *JAMA Pediatrics.*

eTable 2. Summary of Studies Comparing BCG in Preterm/Low-Birthweight Infants to Full-term/Normal-birthweight Infants. Nearly all studies compared early vaccination of both comparator groups; those that included delayed vaccination were Cebeci et al,[43] 2017; Sedaghatian et al,[33] 2009; Negrete-Esqueda et al,[52] 2007; Camargos et al,[42] 2006; Roth et al,[14] 2004; Garly et al,[47] 2003; Sedaghatian et al,[22] 1998; Mussi-Pinhata et al,[20] 1993.

**Studies showing no differences in outcomes between PT and FT; or LBW and NBW infants**					
**Citation**	**Study group**	**Study type**	**Outcome**	**Key Result**	**Comments**
Faridi et al,[44] 2009<br><br>India	143 neonates. 53 PT (31-36 gw) including 50 LBW infants. 90 FT including 28 LBW infants. All vaccinated within 7 days of birth.<br><br>TST (1 TU of PPD) at 12 weeks of postnatal age. LMIT if TST negative. Tests repeated at 6months postnatal age if negative.	Prospective observational study	TST or LMIT positivity at 6 months (combined outcome)<br><br>BCG scar/ reaction presence at 12 weeks	PT: 93%, FT 98%<br>LBW 94%, NBW 99%<br><br>PT: 93%, FT: 89%.<br>LBW 77%, NBW 97%<br>No significant differences between groups for either outcome	LBW and PT neonates had poorer responses to each of TST or LMIT, particularly at earlier time points. Tuberculin reactivity and LMIT continue to increase from 3 to 6 months.<br><br>Strain- BCG Danish 1331 prepared in India
Hawkridge et al,[49] 2008<br><br>South Africa	11 680 newborns randomized to percutaneous versus intradermal BCG at birth. Both trial arms included a total of n=2419 LBW (<2.5kg) and n=2019 PT (<37 gw) infants. 1057 infants were both PT and LBW.	Randomized controlled trial.	Safety<br><br>TB incidence	Keloid scar:_LBW: 2/2419, NBW: 14/9192. Relative Risk: 0.54 (0.12-2.4)<br><br>Suppurative lymphadenitis:_LBW, 3/2419; NBW, 2/9192. Relative Risk: 5.7 (0.95-34)<br>No adverse reactions in PT infants. No cases of disseminated BCG in PT or LBW infants.<br><br>See main text	3-month active follow-up of 4851 infants for adverse reactions. Infants with possible TB infection, morbidity/mortality were passively followed up.<br><br>Unadjusted analyses.<br><br>Strain- BCG Tokyo 172

© 2018 Badurdeen S et al. *JAMA Pediatrics.*

Citation	Study group	Study type	Outcome	Key Result	Comments
Negrete-Esqueda et al,[52] 2007<br><br>Mexico	Group I (GI) included 50 PTIs from 30 to 34.2 gw, vaccinated at 31.4 to 34.6 gw. Group II (GII) included 50 FTIs.<br><br>Evaluated at 4, 8, and 12 weeks after BCG for scar measurement. Sixteen weeks after BCG, the TST was performed.	Prospective cohort	TST, scar<br><br>Safety	The proportion of positive TST in GI (81%) and GII (86%), and the mean TST reaction in GI (6.7 mm) and GII (6.8 mm) were not statistically different (p = 0.74).<br>The percentage of positive scar (GI, 64%; GII, 80%; p = 0.142) and the mean scar diameter (4.5 and 5 mm, respectively) were not statistically significantly different.<br><br>Complications were not observed.	Consecutively allocated by convenience in two groups. 16% loss to follow- up in each group.<br><br>BCG dose 0.05 mL for PTI's, 0.1mL for FTIs.<br><br>Strain- Pasteur-Merieux-Connaught<br>Laboratory, Lyon, France.
Roth et al,[14] 2004<br><br>Guinea-Bissau	Data on TST for 297 children and BCG scar for 1319 children in the study population were re-analyzed for differences between NBW and LBW children.	Retrospective cohort	TST, scar	No significant differences for early BCG in LBW vs NBW:<br><br>TST positivity prevalence ratio 1.39 (95% CI 0.61-3.19), BCG scar positivity prevalence ratio 1.99 (95% CI 0.73-5.40)<br><br>No significant differences for delayed BCG in LBW vs NBW:<br><br>TST positivity prevalence ratio 0.9 (95% CI 0.58-1.38), BCG scar positivity prevalence ratio 0.6 (95% CI 0.29-1.21)	Different TST method used and reaction considered positive if diameter >2mm.<br><br>LBW babies of unknown gestation<br><br>Strain- not stated
Garly et al,[47] 2003<br><br>Guinea-Bissau	2082 infants from 2 cohorts that received BCG (median BCG vaccination age was 9.0 days, inter-quartile range 2–24 days), were recruited at 6 months of age.<br><br>BCG scar and TST response were examined for association	Prospective cohort	Mortality<br><br>TST, scar	10/125 LBW versus 57/1002 NBW infants died. LBW was not an independent risk factor for mortality in univariate or multivariate analyses.<br><br>The authors found no association between birth weight and presence of BCG scar or positive TST. TST	Approximately half of all deaths and just over half of survivors had birthweight data.<br><br>Strain- Pasteur Mérieux, France and SSI, Denmark

	with survival in the following 18+ months, along with other covariates including birthweight.<br><br>BCG often delayed in LBW babies.			was positive in: 276/463 infants (59.6%) with BW $\geq$ 2.5 kg, and 34 /63 infants (54%) with BW < 2.5 kg (univariate OR 0.8, 95% CI: 0.47 - 1.35)	
**Citation**	**Study group**	**Study type**	**Outcome**	**Key Result**	**Comments**
Kaur et al,[50] 2002<br><br>India	90 FT infants and 53 PT infants (31-36 gw) received BCG vaccine within 7 days of life. Of these 78 were LBW.<br><br>The infants were followed up at 4, 6, 8, 10 and 12 weeks to observe reactions at the BCG vaccination site.	Prospective cohort	Reactogenicity and safety	At 8 weeks, BCG reaction was observed in 94% PT, 97% FT, 94% LBW and 98% NBW infants.<br><br>At 12 weeks, 45% of PT, 49% of FT, 44% LBW and 50% NBW infants had a BCG scar (no significant differences between groups).<br><br>Axillary lymph node enlargement was not significantly different between groups.	Strain- BCG Danish 1331 prepared in India
Sedaghatian et al,[22] 1998 UAE	70 PT infants vaccinated at birth, 30 PT infants at 40 weeks and 80 FT infants at birth. PT= 27-36 gw. All neonates were AGA. Sick babies were not excluded.<br><br>TST (10 TU of PPD) done 2-4 months post vaccination.	Randomized trial	TST, scar	No difference in TST induration or BCG scar between any groups. Birthweight significantly associated with a reactive TST in multivariable logistic regression model (OR= 6.2, 95% CI 1.9-20.6)	Very large losses to follow up.<br><br>Strain- lyophilized BCG, Behring laboratories, Germany
Ferreira et al,[45] 1996<br><br>Brazil	Specific immune response to PPD was assessed in 30 LBW newborns (mean birth weight 2311.7 ± 122.1 g; mean gestational age = 38.1 ± 1.8 weeks) in comparison to 56	'Randomized' prospective study	Lymphocyte proliferation, IL-2 production,<br><br>TST, scar	No significant differences between LBW and controls for in vitro lymphocyte proliferation (P = 0.72) or IL-2 production (P = 0.38).	Large losses to follow-up for TST evaluation

© 2018 Badurdeen S et al. *JAMA Pediatrics.*

	control infants (mean birthweight = 3198.9 ± 267.2 g; mean gestational age = 38.5 ± 1.2 weeks).<br><br>Vaccinated at <5d age. Assays (TST with 5 TU of PPD) performed at 12-24 weeks after vaccination.		Safety	TST was also comparable in both groups (mean induration diameter = 9.5 ± 5.1 mm vs 9.6 ± 5.0 mm, P = 0.94). All infants formed a scar.<br><br>No infant presented undesirable local or systemic reactions during the study.	'Randomization' unclear- may have related to inclusion of participants in the study<br><br>Strain- BCG Moreau-Rio de Janeiro
**Citation**	**Study group**	**Study type**	**Outcome**	**Key Result**	**Comments**
Verma et al,[60] 1995<br><br>India	240 LBW and NBW controls (number not stated). All were given BCG within the first week of life.<br><br>Serial tuberculin testing was done at 3, 6, 12, 24, 30, and 36 months of age. The dose of PPD used was 5 TU.	Prospective cohort	TST	No significant differences in diameter at all time points.<br><br>At 36 months mean TST induration was 4.3mm in LBW and 3.7mm in NBW infants	No description of birthweights, gestational ages or other characteristics of study groups.<br><br>BCG strain not stated
Mussi-Pinhata et al,[20] 1993<br><br>Brazil	57 FT infants with symmetric IUGR (<5th centile, 1710-2400g, median 2170 g) and 52 FT AGA infants (10th-90th centile).<br><br>BCG vaccine within 5 days of birth, at 3 months or 6 months of age.<br><br>TST (5 TU of PPD) and LTT performed 12-14 weeks post vaccination	Randomized trial	LTT, TST and scar<br><br>Safety	There were no significant differences in TST diameter or LTT indices between FT IUGR and FT AGA infants vaccinated at similar ages at all time-points.<br>All babies had a BCG scar at 1-2 months.<br><br>None of the 109 infants had any adverse reaction up to 3 months post vaccination.	Strain- Moreau, Rio de Janeiro

© 2018 Badurdeen S et al. *JAMA Pediatrics.*

Studies showing significant differences in outcomes between PT and FT; or LBW and NBW infants					
**Citation**	**Study group**	**Study type**	**Outcome**	**Key Result**	**Comments**
Cebeci et al,[43] 2017<br><br>Turkey	65 PT ≤ 32 gw, 79 between 33-36 gw, 72 FT. All vaccinated at postnatal age 11w ± 3w. Mean weight at BCG vaccination was 4442±1084g.<br><br>TST (5 TU) done 8-16 weeks after vaccination	Prospective observational study	TST<br><br>Scar	TST response rates were 67%, 55%, and 47% according to GA ≥ 37 w, 33 - 36 w, and ≤ 32 w, respectively (P = 0.020).<br>TST response rates were 80%, 54%, and 50% according to birth weight > 2500g, 1500-2500, and < 1500g, respectively (P = 0.213).<br><br>GA, birth weight, weight appropriate for GA, or corrected age at the time of BCG vaccination were not significantly associated with BCG scar formation.	BCG strain not stated
Timmermann et al,[34] 2015<br><br>Guinea-Bissau	2709 normal birthweight and 1102 neonates <2.5kg that were randomized to BCG at birth in 2 separate RCT's.<br><br>Examined for scar and TST (0.1ml PPD) response at 2 and 6 months.	Prospective cohorts	TST<br><br>Scar	2months: LBW: 17%, NBW 36%. aOR: 0.35 (0.17- 0.75)<br>6months: LBW: 28%, NBW 35%<br><br>2months: LBW: 91%, NBW 88%<br>6months: LBW: 93%, NBW 93%	LBW cohort here is the 'early' BCG group of the RCT by Aaby et al. 2011.[37]<br><br>Only study to adjust for potential confounders. Odds ratios at other timepoints not provided.<br><br>Strain- BCG Danish 1331 (SSI)
Sartono et al,[56] 2010<br><br>Guinea-Bissau	250 LBW and 540 NBW infants who had been given early BCG, some of whom received concurrent oral polio vaccine.	Retrospective cohort study	TST & scar at 2 and 6 months	At 2 months, 13 % of LBW and 37% of NBW babies were TST positive (RR 0.35, CI 0.23-0.51)<br>At 6 months, this was 26% Vs 33 % respectively (RR 0.79, 0.58-1.07)<br><br>Prevalence of scarring was very similar in both cohorts at both timepoints (92-96 %)	Unadjusted analysis. We combined infants who did and did not receive oral polio vaccine in each of the LBW and NBW groups.<br><br>The median cytokine level in the total population was

© 2018 Badurdeen S et al. *JAMA Pediatrics.*

	Post-hoc analysis of data from 2 separate concurrently-conducted trials.<br><br>61 LBW and 248 NBW infants had cytokine profiles.<br><br>TST done using 0.1ml PPD at 2 and 6 months of age		*In-vitro* whole blood cytokine (IFN-γ and TNF-α) responses to PPD at 6 weeks age	Percentage of 'high responders' in LBW infants vs NBW infants:<br>IFN-γ: 59% vs 48%<br>TNF-α: 54% vs 49%	used as the cut off for low and high responders<br><br>Strain- BCG Danish 1331 (SSI)
**Citation**	**Study group**	**Study type**	**Outcome**	**Key Result**	**Comments**
Dube et al,[61] 2009<br><br>Abstract<br><br>South Africa	Infants who received routine BCG vaccination at birth were stratified for BW and for GA.<br><br>At 10 weeks of age, whole blood was collected and incubated with viable BCG. White cells were stained for CD3, CD4, CD8, IFN-γ, TNF-α, IL-2, and IL-17; expression of these markers was measured by multi-parameter flow cytometry.	Prospective cohort	Cytokine expression	LBW infants (n=52) had a lower proportion of BCG-specific CD4 T cells co-expressing IFN-γ, TNF-α and IL-2 together, compared with infants with normal BW (n=53; 16.9+/-1.1% vs. 21.3+/-11.2%, respectively, p=0.01), and a higher proportion of CD4 T cells expressing IFN-γ only (33.3+/-1.9% vs. 27.6+/-1.5%, p=0.02). No other differences in cytokine expression were shown.	BCG strain not stated
Sedaghatian et al,[33] 2009<br><br>UAE	Fifty-two PT infants (27-36 gw) and 31 FT infants received BCG at birth. A further 29 PTI's were vaccinated at term.<br><br>TST and LTT were performed 2-4months after vaccination, and BCG scar presence was noted.	Prospective cohort	LTT, TST and BCG scar	LTT responses, but not TST or scar rates, were significantly better in FTIs when compared to PTIs either vaccinated at birth (P=0.0001) or near term (P=0.001).	Large losses to follow up<br><br>Strain- Merieux seed 1077 strain

© 2018 Badurdeen S et al. *JAMA Pediatrics.*

Citation	Study group	Study type	Outcome	Key Result	Comments
Camargos et al,[42] 2006<br><br>Brazil	182 FT and 71 PT newborns. PT infants (32-36 gw) were vaccinated when their body weight reached >1900g.<br><br>Unwell babies were excluded.<br><br>TST (0.1ml PPD-S) was performed blinded to gestational age at birth and within 3 months after vaccination.	'Concurrent cohort' study, with inclusion of participants randomized	TST, scar<br><br><br><br>Safety	TST positive in 88% of FT (n=65) and 68% of the PT infants (n=40, P = 0.02).<br><br>BCG scar present in 97% of FT and in 90% of the PT infants (P = 0.19).<br><br>No adverse reactions were observed in either group	Randomization procedures not specified.<br><br>Large losses to follow up both in FT (46%) and PT (44%) groups<br><br>Strain- BCG Moreau-Rio de Janeiro
Osendarp et al,[54] 2006<br><br>Bangladesh	405 infants were vaccinated with BCG within 72 hours after birth, of which 43% were LBW.<br><br>TST done at 24 weeks of age (0.1ml PPD)	RCT of Zn supplementation during pregnancy.<br><br>TST used as a marker for cell-mediated immune response.	TST	A significantly higher percentage of LBW infants showed negative skin responses compared to NBW infants (71.9% vs. 52.7% respectively, P<0.0001).<br><br>No differences in the mean size of PPD induration in positive responders	LBW and NBW groups reported here include infants who also received concurrent Zn supplementation<br><br>50 infants followed up were PT.<br><br>BCG strain not stated
Roth et al,[55] 2005<br><br>Guinea Bissau	3905 infants were followed up at 2, 6 and 12 months of age. Varying numbers had TST (0.1ml PPD) and scar assessment at each visit.<br><br>Approximately 85% had BCG under 1 month old, and 97% before 5 months.<br><br>Reactors to PPD (>1 mm) and non-reactors to PPD (0–1 mm).	Retrospective cohort	TST and BCG scar at 6 months	149 of 177 LBW babies had a scar compared with 1160 of 1289 NBW babies; aOR 0.42 (95% CI 0.24-0.73) p=0.002.<br><br>LBW had no impact on TST	Large numbers joined or left the cohort during the study. Birthweight parameter was only available for the 69% of the children.<br><br>Three types of BCG vaccines were used, Pasteur Merieux (France), Connaught (Canada) and SSI (Denmark)

© 2018 Badurdeen S et al. *JAMA Pediatrics.*

Citation	Study group	Study type	Outcome	Key Result	Comments
Tipayamong-khogul et al,[58] 2005<br><br>Thailand	130 children <15 years with TB, compared with 130 age and sex-matched controls. Biological, socioeconomic and environmental data collected by questionnaire, before uni- and multi-variate analysis.	Case-control	Efficacy (TB disease)	15 of 126 cases were LBW (12%), compared with 11 of 130 controls (9%). Reported age-adjusted univariate OR 1.47 (95% CI: 0.65-3.33).	Timing of BCG is implied to be 'at birth'<br><br>BCG strain not stated
Tun et al,[59] 2000<br><br>Myanmar	116 FT infants were vaccinated at birth, 89 FT infants at the end of the 3rd month of life and 48 PT infants (32-36 gw) at birth.<br><br>Follow-up and TST (2 TU) at 3 months chronological age	Trial	TST, scar<br><br>Safety	PPD >5mm: 13/48 of PT vs 81/116 FT infants at birth. PPD induration mean difference 3.8mm (95% CI 2.77-4.84)<br>No scar: 10% of PT vs 4% of all FT babies<br><br>None of the infants had any complication resulting from the BCG vaccine on follow-up.	Method used for recruitment was not stated. No blinding.<br><br>Strain- BCG Japan
Neumann et al,[53] 1998<br><br>Kenya	IUGR newborns: group 1 severe (<3rd centile, <2500g, n=16); group 2 moderate (3rd-10th centile, 2501-2799 g, n=27). These included 7 PT 33-37 weeks. Group 3 term AGA controls (n=26).<br><br>BCG given at birth. 5 TU of PPD at 6 Months of Age.	Prospective cohort	TST	Mean induration for Groups I, II and III respectively were 5.3 mm, 10.6 mm and 11.3 mm with significantly less induration in Group I compared to Group III (p<0.025).	Method used for recruitment was not stated.<br><br>BCG strain not stated
Sepulveda et al,[57] 1994<br><br>Chile	134 infants had BCG at birth or once they reached 2000g. Identified from a newborn registry.<br><br>TST (2 TU of PPD) at 3-34 months of age.	Cohort	TST	Regression analyses suggested that BW was significantly associated with TST reaction; parameter estimate -6.7 (P = 0.04)	70 of these infants were twins<br><br>Strain- BCG Japan

© 2018 Badurdeen S et al. *JAMA Pediatrics.*

Citation	Study group	Study type	Outcome	Key Result	Comments
Grindulis et al,[48] 1984<br><br>UK	149 children with South Asian mothers received BCG vaccine 'shortly after birth'.<br><br>TST (10 TU of PPD) was performed at 21-21 months of age	Retrospective cohort	TST, scar	112 (75%) had a scar. 69 (53%) had a positive TST.<br><br>No clear association of either outcome with gestational age or birth weight. However, Small for Gestational Age (SGA) babies were less likely to develop a scar (P<0.05).	LBW babies were included in the parent trial, but exact weights are not stated<br><br>Strain- not stated
Manerikar et al,[51] 1976<br><br>India	44 FT neonates: 14 with severe-IUGR (<0.3rd centile), 10 with mild-IUGR (0.3rd-10th centile) and 20 AGA controls. All received BCG within 48h of birth.<br><br>TST with 1 TU of PPD. LMIT at 8 weeks postnatal age.	Prospective cohort	TST, LMIT	TST conversion: s-IUGR 1/14, significantly different to m-IUGR 5/10 and controls 12/20. P<0.05<br><br>Positive LMIT: s-IUGR 1/14, significantly different to m-IUGR 6/10 and controls 12/20. P<0.05	Timing of TST not stated.<br><br>Strain- BCG Laboratories, Guindy, Madras
Gaisford,[46] 1955<br><br>UK	5215 newborn infants over 5 years given BCG, followed up annually. Little data on loss to follow up. Included "a number of premature infants" of $\geq$1.8 kg.	Prospective cohort	Safety, efficacy	"No adverse systemic effects in any of the premature infants but an increased incidence of adenitis" (data not shown). No cases of TB death or disease in either FT or PT babies.	If 5-10% of births were PT, this would constitute approximately 250-500<br><br>Strains- BCG Danish (>80%), French, vole and Swedish.

Abbreviations: AGA, appropriate for gestational age; aOR, adjusted odds ratio; CI, confidence interval; FT, full term; GA, gestational age; gw, gestational weeks; IUGR, intrauterine growth retardation; LBW, low birthweight; LMIT, lymphocyte migration inhibition test; LTT, lymphocyte transformation test; NBW, normal birthweight; PT, preterm; RCT, randomized controlled trial; SGA, Small for Gestational Age; SSI, Statens Serum Institut; TST, tuberculin skin test; TU, tuberculin units

© 2018 Badurdeen S et al. *JAMA Pediatrics.*
